# Ketamine Compared With Morphine for Out-of-Hospital Analgesia for Patients With Traumatic Pain: A Randomized Clinical Trial

**DOI:** 10.1001/jamanetworkopen.2023.52844

**Published:** 2024-01-29

**Authors:** Clément Le Cornec, Marion Le Pottier, Hélène Broch, Alexandre Marguinaud Tixier, Emmanuel Rousseau, Said Laribi, Charles Janière, Vivien Brenckmann, Anne Guillerm, Florence Deciron, Amine Kabbaj, Joël Jenvrin, Morgane Péré, Emmanuel Montassier

**Affiliations:** 1 Department of Emergency Medicine, Centre Hospitalier Universitaire (CHU) Nantes, Nantes, France; 2 Département de Médecine d’Urgences, CHU Angers, Angers, France; 3 Urgences Service Mobile d’Urgence et de Réanimation (SMUR), Centre Hospitalier Chateaubriant, Chateaubriant, France; 4 Pôle Urgences Adultes–Service d’Aide Médicale Urgente (SAMU), Hôpital Pellegrin, CHU de Bordeaux, Bordeaux, France; 5 Department of Emergency Medicine, CHU Rennes, Rennes, France; 6 Centre Hospitalier Régional et Universitaire Tours Urgences SAMU 37 SMUR de Tours, Tours, France; 7 SAMU85 Centre Hospitalier Départemental Vendée la Roche sur Yon, la Roche sur Yon, France; 8 Urgences SAMU CHU Grenoble Alpes, Grenoble, France; 9 SMUR Centre Hospitalier Gonesse, Gonesse, France; 10 Centre Hospitalier Le Mans SAMU 72 SMUR du Mans, Le Mans, France; 11 Centre Hospitalier Saint Nazaire Urgences SMUR de Saint Nazaire, Saint Nazaire, France; 12 Plateforme de Méthodologie et Biostatistique, CHU Nantes, Nantes, France; 13 Center for Research in Transplantation and Translational Immunology, Unité Mixte de Recherche 1064, Nantes Université, CHU Nantes, Institut National de la Santé et de la Recherche Médicale, Nantes, France

## Abstract

**Importance:**

Pain is a common out-of-hospital symptom among patients, and opioids are often prescribed. Research suggests that overprescribing for acute traumatic pain is still prevalent, even when limits restricting opioid prescriptions have been implemented. Ketamine hydrochloride is an alternative to opioids in adults with out-of-hospital traumatic pain.

**Objective:**

To assess the noninferiority of intravenous ketamine compared with intravenous morphine sulfate to provide pain relief in adults with out-of-hospital traumatic pain.

**Design, Setting, and Participants:**

The Intravenous Subdissociative-Dose Ketamine Versus Morphine for Prehospital Analgesia (KETAMORPH) study was a multicenter, single-blind, noninferiority randomized clinical trial comparing ketamine hydrochloride (20 mg, followed by 10 mg every 5 minutes) with morphine sulfate (2 or 3 mg every 5 minutes) in adult patients with out-of-hospital trauma and a verbal pain score equal to or greater than 5. Enrollment occurred from November 23, 2017, to November 26, 2022, in 11 French out-of-hospital emergency medical units.

**Interventions:**

Patients were randomly assigned to ketamine (n = 128) or morphine (n = 123).

**Main Outcomes and Measures:**

The primary outcome was the between-group difference in mean change in verbal rating scale pain scores measured from the time before administration of the study drug to 30 minutes later. A noninferiority margin of 1.3 was chosen.

**Results:**

A total of 251 patients were randomized (median age, 51 [IQR, 34-69] years; 111 women [44.9%] and 140 men [55.1%] among the 247 with data available) and were included in the intention-to-treat population. The mean pain score change was −3.7 (95% CI, −4.2 to −3.2) in the ketamine group compared with −3.8 (95% CI, −4.2 to −3.4) in the morphine group. The difference in mean pain score change was 0.1 (95% CI, −0.7 to 0.9) points. There were no clinically meaningful differences for vital signs between the 2 groups. The intravenous morphine group had 19 of 113 (16.8% [95% CI, 10.4%-25.0%]) adverse effects reported (most commonly nausea [12 of 113 (10.6%)]) compared with 49 of 120 (40.8% [95% CI, 32.0%-49.6%]) in the ketamine group (most commonly emergence phenomenon [24 of 120 (20.0%)]). No adverse events required intervention.

**Conclusions and Relevance:**

In the KETAMORPH study of patients with out-of-hospital traumatic pain, the use of intravenous ketamine compared with morphine showed noninferiority for pain reduction. In the ongoing opioid crisis, ketamine administered alone is an alternative to opioids in adults with out-of-hospital traumatic pain.

**Trial Registration:**

ClinicalTrials.gov Identifier: NCT03236805

## Key Points

**Question:**

Is intravenous ketamine hydrochloride noninferior to intravenous morphine sulfate in adults with out-of-hospital traumatic pain?

**Findings:**

In this randomized clinical trial that included 251 patients, the mean pain score change at 30 minutes was −3.7 in the ketamine group compared with −3.8 in the morphine group, a difference of 0.1 that met criteria for noninferiority.

**Meaning:**

These findings show that ketamine was not inferior to morphine for pain control and is an opioid-reduction alternative for treatment of out-of-hospital acute traumatic pain.

## Teaser

This randomized clinical trial compares intravenous ketamine and morphine in patients with out-of-hospital traumatic pain.

## Introduction

Pain is a common out-of-hospital symptom and inadequate pain management is frequent in patients with traumatic pain.^1,2,3^ There is considerable debate about optimal out-of-hospital analgesia in patients with traumatic pain and wide variation in analgesic drugs prescribed by prehospital care clinicians.^4^ Opioids are often prescribed, but opioid prescription is associated with severe adverse events, including respiratory depression, hypotension, bradycardia, and oversedation.^5,6,7^ Opioids are also highly addictive, and some patients may develop dependence, even when exposed to short-term treatment administered for pain relief.^1,2,8,9,10^ In the US, overprescription of opioids has been a key factor in rising overdoses and death rates.^8,11^ Research has highlighted that overprescribing for acute traumatic pain is still prevalent, even when limits restricting the duration or number of doses in opioid prescriptions for acute pain are implemented.^12^

Changing prescribing practices is a critical step in addressing the opioid epidemic. In an out-of-hospital setting, opioid-reduction strategies should be implemented to manage acute pain.^13^ Ketamine hydrochloride is commonly used at a dissociative dose for procedural sedation.^14^ At a subdissociative dose, most commonly 0.3 mg/kg, ketamine provides analgesic effects.^15^ What makes ketamine attractive is that it allows patients to maintain their pharyngeal reflexes and own airways. A recent systematic review concluded that ketamine probably reduces pain more than opioids, with less nausea and vomiting but with a higher risk of agitation.^4^ However, the included studies were heterogeneous in terms of settings, patient populations, outcomes, and comparators.^4^

Given the lack of high-quality evidence, we conducted a randomized clinical trial to compare intravenous ketamine vs morphine sulfate in patients with out-of-hospital trauma. We hypothesized that ketamine would be noninferior to morphine for pain relief.

## Methods

### Study Design

The Intravenous Subdissociative-Dose Ketamine Versus Morphine for Prehospital Analgesia (KETAMORPH) study was a prospective, multicenter, single-blind, noninferiority randomized clinical trial to compare the effect of intravenous ketamine with that of intravenous morphine in the treatment of adults with out-of-hospital traumatic pain. This study, performed between November 23, 2017, and November 26, 2022, involved 11 out-of-hospital emergency medical services (EMS) centers in France. These centers are ambulance base stations equipped with 1 or more mobile intensive care units consisting of an ambulance driver, a nurse, and an emergency physician as the minimum team.^16^ The participants were blinded to the study arm in which they were enrolled but the physicians conducting the out-of-hospital pain management were not blinded for the following reasons: (1) patients receiving ketamine were expected to exhibit obvious and easily identifiable effects, making the study arm allocation obvious to the clinician and negating the intent of masking; (2) the need to double-check drugs and doses according to standard operating procedures to ensure patient safety; and (3) the primary outcome was assessed by the patient using the verbal rating scale without any possible intervention of the physician in charge of the patient, contrary to earlier research where the verbal rating scale ratings were measured by the physicians who administered the analgesic.^17^

The Comité de Protection des Personnes Sud-Méditerranée II ethics committee approved the trial protocol. Patients with out-of-hospital trauma and with severe pain are most often not able to provide informed verbal or written consent, because they need urgent pain management and because acute pain interferes with the ability to provide informed consent. Whenever a patient was included without written informed consent, such consent was promptly sought, according to the French Law of Ethics, from the patient when the pain decreased. The trial protocol is available in Supplement 1; patient consent and case report form completion details are provided in the eMethods in Supplement 2. The study followed the Consolidated Standards of Reporting Trials (CONSORT) reporting guideline.

### Patient Population

Patients were eligible for enrollment if the attending EMS determined that they met the following criteria: aged 18 years or older, conscious (Glasgow Coma Scale score of 15 [range, 3 [worst] to 15 [best]), reporting acute traumatic pain with a verbal numeric rating scale pain score of 5 or greater on a standard 11-point numeric rating scale (where 0 indicates no pain and 10, worst possible pain), speaking, and being able to rate their pain with the verbal numeric rating scale. Patients were excluded if any of the following applied: unstable vital signs (systolic blood pressure <90 or >200 mm Hg, pulse rate <50 or >150 beats/min, and respiration rate <10 or >30 breaths/min), pregnancy, breast-feeding, unable to rate their pain with the verbal numeric rating scale scores, allergy to morphine or ketamine, acute pulmonary edema or acute heart failure, acute coronary syndrome or unstable ischemic heart disease, renal or hepatic insufficiency, receiving morphine for the same acute pain or acute psychiatric illness, requiring emergency fracture or joint reduction, head injury with acute intracranial hypertension, or receiving buprenorphine hydrochloride, nalbuphine hydrochloride, pentazocine hydrochloride, or naltrexone hydrochloride.

### Study Intervention

**Figure 1.**
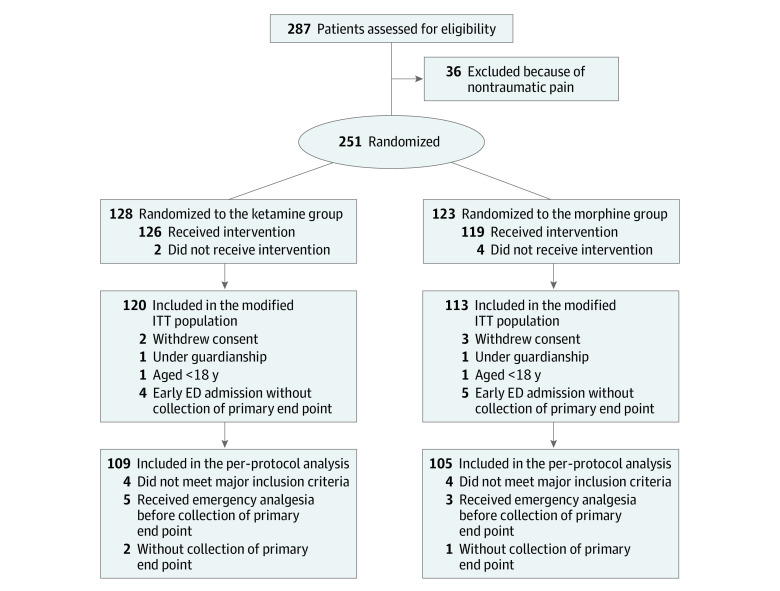
Study Flow Diagram Adapted from the 2010 CONSORT guidelines. ED indicates emergency department. ITT indicates intention to treat.

Patients were randomized in a 1:1 ratio to the ketamine or the morphine group (Figure 1). A computerized random number generator created the randomization list (1:1). Central randomization was defined without block but was stratified by center. Group assignments were then sent in sealed envelopes to the study centers.

Ten milligrams of morphine sulfate were diluted in 9 mL of normal saline solution, resulting in a 1-mg/mL solution. Morphine sulfate was administered by intravenous push, 2 mg (patient weight <60 kg) or 3 mg (patient weight ≥60 kg) every 5 minutes.^18^ Two hundred milligrams of ketamine hydrochloride was diluted in 18 mL of normal saline solution, resulting in a 10-mg/mL solution. Ketamine hydrochloride was administered by intravenous push of 20 mg over 2 minutes followed by 10 mg every 5 minutes.^18^ Emergency physicians used their clinical judgment on dosing according to patient age and body size. Either morphine or ketamine continued to be administered according to this schedule until the patient obtained pain relief (numeric rating scale pain score ≤3),^19^ there was a serious adverse event (ie, profound hypotension, unconsciousness, respiratory depression requiring ventilatory support), or the patient arrived at the receiving emergency department (ED). When pain relief was not achieved despite multiple doses of both analgesics, rescue analgesia was administered to the patient for additional pain relief. The choice of drugs and dose was left to the discretion of the emergency physician.^20^ For patients with a blood oxygen saturation level below 94% during the administration of the study drug, oxygen was administered via a nasal cannula with a delivering flow rate of 2 L/min and was adapted based on blood oxygen saturation level on follow-up.

### Outcomes

The primary outcome measure was the between-group difference in mean change in verbal numeric rating scale pain scores among patients receiving ketamine or morphine, measured from the time before administration of the study medication to 30 minutes later. Secondary prespecified outcomes were (1) between-group difference in the mean change in numeric rating scale pain scores among patients receiving ketamine or morphine measured from the time before administration of the study medication to 15, 45, and 60 minutes later and on ED admission; (2) the incidence of rescue analgesia; (3) the change in vital signs at 15, 45, and 60 minutes and on ED admission; (4) the incidence of adverse events; (5) the need to withdraw morphine or ketamine analgesia and the use of specific drugs to antagonize severe adverse events; and (6) the weight-based dose of the study drug (in mg/kg) received during the 30-minute period. We actively sought adverse events associated with morphine or ketamine use.^21^ Follow-up ended 24 hours after the last administration of ketamine or morphine for each patient based on the half-time elimination of the study drugs.

### Sample Size

The hypotheses for sample size calculations integrated the results of 3 randomized clinical trials that focused on acute extremity pain in the emergency department,^20,21,22^ including 1 trial using the same active comparator. A reduction of the verbal numeric rating scale pain score of greater than 1.3 was considered clinically meaningful. After assuming a noninferiority margin of 1.3 with a type I error of 5%/2 and type II error of 10%, we determined that 224 patients were needed (112 in each treatment group). We set targeted enrollment at 248 patients to account for the risks of protocol deviations, considering that 10% of patients may not be evaluable. We therefore planned to include 124 patients in each group.

### Statistical Analysis

Characteristics at baseline were described by their frequency (percentages) for categorical variables and by means (SDs) or medians (IQRs) for quantitative variables. The primary aim of the trial was to assess the noninferiority of intravenous ketamine vs morphine with a between-group difference in mean change in verbal rating scale pain scores measured from the time before administration of the study medication to 30 minutes later (π) as the primary end point. Analysis of the primary end point was performed by calculating the 97.5% 1-sided CI of the difference as π ketamine − π morphine. The conclusion of noninferiority would be accepted if the higher limit of this CI was lower than 1.3. Because it was a noninferiority trial, the main analysis was based on both the intention-to-treat (ITT) population of all randomized patients and the per-protocol analysis of all patients randomized and treated without major protocol violations or deviations, following the extension of the CONSORT reporting guideline for randomized controlled trials.^23^

The secondary end points were tested for superiority in the modified ITT population of all randomized patients except for those who withdrew consent to participate, were under guardianship, were younger than 18 years, or were admitted to the ED before 30 minutes. Qualitative secondary end points were analyzed with the χ^2^ test or the Fisher exact test when necessary. Proportions differences and the corresponding 95% CIs were estimated. For quantitative secondary end points, the 2-tailed *t* test or Mann-Whitney test were used according to their Gaussian or non-Gaussian statistical distribution.

A linear mixed model was used for the primary outcome analysis. The analysis was adjusted for center as a random effect and did not include 45-minute, 60-minute, or other covariates. The significance threshold was 2-sided *P* = .05 without adjustment for multiplicity. Analyses used SAS software, version 9.4 (SAS Institute Inc). The detailed statistical analysis plan is available in Supplement 1.

## Results

### Baseline Characteristics

A total of 251 patients were enrolled during the study (median age, 51 [IQR, 34-69] years); of the 247 with data available, 111 were women (44.9%) and 136 were men (55.1%). A total of 128 patients were enrolled in the ketamine group and 123 in the morphine group (Figure 1). The number of inclusions for each investigator center is detailed in eTable 1 in Supplement 2. We included more patients (n = 3) than planned in 1 center because the 3 paper case report forms were recorded but not entered in the online centralized database by the local investigator. The population of the per-protocol analysis consisted of 109 patients in the ketamine group and 105 patients in the morphine group (see reasons for exclusion in Figure 1). Patient characteristics were well balanced between the 2 groups except for history of diabetes and coronary heart disease (Table 1). None of the observed differences appeared to be clinically meinaningful.

**Table 1.** Demographic Data and Injury Characteristics of Patients

Characteristic	Patient group^a^		
	All (N = 251)	Ketamine (n = 128)	Morphine (n = 123)
Sex^b^			
Women	111 (44.9)	59 (46.8)	52 (43.0)
Men	136 (55.1)	67 (53.2)	69 (57.0)
Age, y			
Median (IQR)	51 (34-69)	50 (32-68)	52 (37-70)
Range	16-95	18-95	16-92
Estimated BMI, median (IQR)	24.5 (22.3-27.7)	24.2 (22.3-27.7)	24.5 (22.3-27.7)
Medical history^b^			
Hypertension	62 (25.1)	37 (29.4)	25 (20.7)
Cancer	11 (4.5)	6 (4.8)	5 (4.1)
Neurological disorder	11 (4.5)	6 (4.8)	5 (4.1)
Diabetes	13 (5.3)	9 (7.1)	4 (3.3)
Coronary heart disease	7 (2.8)	7 (5.6)	0
Chronic obstructive pulmonary disease	4 (1.6)	2 (1.6)	2 (1.7)
Chronic kidney failure	1 (0.4)	0	1 (0.8)
Liver disease	1 (0.4)	0	1 (0.8)
Thyroid dysfunction	15 (6.1)	9 (7.1)	6 (5.0)
Psychiatric disorder	8 (3.2)	5 (4.0)	3 (2.5)
Already received opioids^c^	10 (4.0)	5 (4.0)	5 (4.1)
Use of another psychoactive drug	31 (12.6)	12 (9.5)	19 (15.7)
Case nature			
Extremity fracture	102 (40.6)	42 (32.8)	60 (48.8)
Soft tissue injury	95 (37.8)	52 (40.6)	43 (35.0)
Fracture, other	14 (5.6)	10 (7.8)	4 (3.3)
Dislocation	34 (13.5)	19 (14.8)	15 (12.2)
Burn	6 (2.4)	5 (3.9)	1 (0.8)
Injury severity score			
Median (IQR)	4.0 (3.0-5.0)	4.0 (3.0-5.0)	4.0 (3.0-5.0)
Range	1.0-12.0	1.0-12.0	1.0-12.0
Initial pain score			
Median (IQR)	8.0 (7.0-10.0)	8.0 (7.0-10.0)	8.0 (6.5-9.0)
Range	5.0-10.0	5.0-10.0	5.0- 10.0
Dose of trial drug administered after randomization, mg			
Median (IQR)	NA	40.0 (30.0-50.0)	12.0 (8.5-16.0)
Range	NA	15.0-100.0	3.0-50.0
Acetaminophen administration^d^	187 (76.6)	93 (74.4)	94 (79.0)
Characteristics on enrollment			
GCS score, median (IQR)^e^	15.0 (15.0-15.0)	15.0 (15.0-15.0)	15.0 (15.0-15.0)
Heart rate, median (IQR), beats/min	80.0 (70.0-93.0)	80.0 (70.0-93.0)	81.0 (70.0-90.0)
Respiratory rate, median (IQR), breaths/min	18.0 (16.0-20.0)	18.0 (16.0-22.0)	18.0 (16.0-20.0)
Arterial systolic pressure, median (IQR), mm Hg^f^	141.0 (127.0-153.0)	141.0 (125.0-153.0)	140.5 (128.0-154.0)
<90	25 (10.9)	10 (8.5)	15 (13.4)
>140	117 (51.1)	61 (52.1)	56 (50.0)
Arterial diastolic pressure, mm Hg^f^	84.0 (74.0-91.0)	83.0 (73.0-91.0)	84.0 (74.5-90.0)
<50	2 (0.9)	1 (0.9)	1 (0.9)
>90	59 (25.8)	32 (27.4)	27 (24.1)
Peripheral oxygen saturation, median (IQR), %	99.0 (97.0-100.0)	99.0 (97.0-100.0)	99.0 (97.0-100.0)
Out-of-hospital time, min			
Median (IQR)	64.0 (52.0-82.0)	61.0 (52.0-78.0)	67.0 (20.5-85.0)
Range	22.0-134.0	22.0-134.0	26.0-122.0

Abbreviations: BMI, body mass index (calculated as weight in kilograms divided by height in meters squared); GCS, Glasgow Coma Scale.

^a^ Unless otherwise indicated, data are expressed as No. (%) of patients. Percentages have been rounded and may not total 100.

^b^ Data were missing for 4 patients.

^c^ Already received opioids for another episode.

^d^ Data were missing for 7 patients.

^e^ Scores range from 3 (worst) to 15 (best).

^f^ Data were missing for 22 patients.

### Primary Outcome

**Figure 2.**
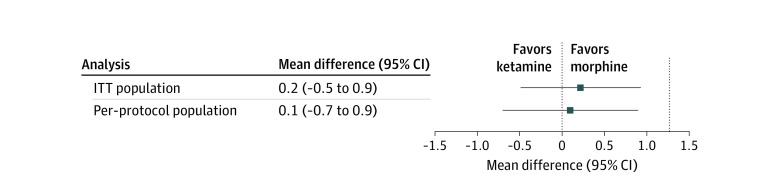
Difference in Pain Between the Ketamine and Morphine Groups Difference was measured as the mean change in verbal rating scale pain scores from the time before administration of the study drug to 30 minutes later by calculating the 97.5% 1-sided CI of the difference as π ketamine − π morphine. The noninferiority margin was defined at 1.3 (dotted vertical line at far right). ITT indicates intention to treat.

In the per-protocol population, the mean change in pain score between drug administration and 30 minutes later was −3.7 (95% CI, −4.2 to −3.2) in the ketamine group compared with −3.8 (95% CI, −4.2 to −3.4) in the morphine group (difference, 0.1 [95% CI, −0.7 to 0.9] points) (Figure 2). The upper limit of the CI was lower than the threshold of noninferiority. Noninferiority was therefore demonstrated. In the ITT population, noninferiority was also demonstrated: the mean pain score change was −3.6 (95% CI, −5.0 to −2.0) in the ketamine group compared with −3.8 (95% CI, −5.0 to −2.0) in the morphine group (difference, 0.2 [95% CI, −0.5 to 0.9]) (Figure 2).

### Secondary Outcomes

The ketamine group had a faster reduction in pain intensity than the morphine group (mean values of pain points per minute: −0.09 [95% CI, −0.10 to −0.08] in the ketamine group and −0.07 [95% CI, −0.08 to −0.05] in the morphine group; difference: 0.02 [95% CI, 0.01-0.04; *P* = .14]) (eFigure in Supplement 2). We did not find any significant difference when we compared the mean pain score change between drug administration and 45 minutes later among patients with pain score data available (ketamine [n = 30], −4.5 [95% CI, −6.0 to −2.0]; morphine [n = 39], −5.00 [95% CI, −7.0 to −4.0]; *P* = .07) and between administration and 60 minutes later (ketamine [n = 5], −4.0 [95% CI, −5.0 to −3.0]; morphine [n = 6], −5.5 [95% CI, −7.0 to −4.0]; *P* = .42) (eFigure in Supplement 2).

Vital signs were assessed on enrollment and at 15-minute intervals thereafter until arrival at the receiving ED. Changes in vital signs during this time are illustrated in Table 2 and eTable 2 in Supplement 2. We did not observe significant differences between the 2 groups. The incidence of rescue analgesia was low and did not differ between the ketamine group (5 of 120 [4.2% (95% CI, 1.4%-9.5%)]) and the morphine group (4 of 113 [3.5% (95% CI, 1.0%-8.8%)]; difference, 0.6% [95% CI, −0.4% to 5.6%]; *P* = .80).

**Table 2.** Vital Sign Changes During Out-of-Hospital Management for Pain by Study Group

Parameter^a^	Patient group		Risk difference^b^	*P* value
	Ketamine (n = 120)	Morphine (n = 113)		
Pulse rate, mean (SD), beats/min				
T0	81.7 (16.7)	81.7 (16.2)	NA	NA
T15	82.6 (17.0)	79.2 (15.4)	NA	NA
Mean change (95% CI)	0.85 (−1.3 to 3.0)	−2.6 (−4.7 to −0.4)	3.4 (0.3 to 6.5)	.03
T30	80.1 (16.9)	78.6 (15.5)		
Mean change (95% CI)	−1.8 (−4.2 to 0.7)	−3.5 (−6.0 to −1.0)	1.8 (−1.8 to 5.3)	.33
T45	80.7 (16.4)	78.1 (14.7)	NA	NA
Mean change (95% CI)	−2.0 (−14.3 to 10.3)	−8.2 (−13.7 to −2.8)	3.8 (−4.7 to 16.3)	.09
T60	80.4 (16.5)	74.0 (12.1)	NA	NA
Mean change (95% CI)	3.6 (−21.1 to 28.3)	−6.7 (−25.1 to 11.7)	NA	NA
Respiratory rate, median (IQR), breaths/min				
T0	18.0 (16.0 to 22.0)	18.0 (16.0 to 20.0)	NA	NA
T15	18.0 (16.0 to 22.0)	18.0 (16.0 to 20.0)	0.6 (−0.7 to 1.9)	.34
Mean change (95% CI)	−0.7 (−1.6 to 0.2)	−1.3 (−2.3 to −0.4)	NA	NA
T30	18.0 (15.0 to 21.0)	17.0 (15.0 to 18.0)	NA	NA
Mean change (95% CI)	−1.2 (−2.1 to −0.2)	−2.0 (−3.1 to −1.0)	0.9 (−0.6 to 2.3)	.24
T45	20.0 (20.0 to 24.0)	16.0 (13.0 to 18.0)	NA	NA
Mean change (95% CI)	−1.0 (−2.8 to 0.7)	−4.2 (−6.5 to −1.9)	3.1 (0.2 to 6.1)	.04
T60	16.0 (16.0 to 22.5)	18.0 (14.0 to 20.0)	NA	NA
Mean change (95% CI)	−2.5 (−11.7 to 6.7)	−4.5 (−17.7 to 8.7)	NA	NA
Systolic blood pressure, mean (SD), mm Hg				
T0	143.2 (26.8)	141.4 (20.4)	NA	NA
T15	146.8 (26.8)	137.7 (22.3)	NA	NA
Mean change (95% CI)	3.2 (−1.1 to 7.4)	−3.4 (−6.6 to −0.2)	6.5 (1.2 to 11.9)	.02
T30	143.1 (21.2)	135.4 (22.2)	NA	NA
Mean change (95% CI)	−0.6 (−5.2 to 4.0)	−6.4 (−10.2 to −2.6)	5.8 (−0.2 to 11.8)	.06
T45	147.6 (22.6)	134.2 (17.6)	NA	NA
Mean change (95% CI)	−2.0 (−14.2 to 10.3)	−8.3 (−13.7 to −2.8)	6.2 (−6.0 to 18.5)	.31
T60	147.0 (29.8)	130.4 (20.9)	NA	NA
Mean change (95% CI)	3.6 (−21.1 to 28.3)	−6.7 (−25.1 to 11.7)	NA	NA
Diastolic blood pressure, mean (SD), mm Hg				
T0	84.4 (18.0)	83.5 (13.8)	NA	NA
T15	88.1 (16.9)	82.7 (17.8)	NA	NA
Mean change (95% CI)	3.6 (0.8 to 6.5)	−1.0 (−4.5 to 2.5)	4.6 (0.2 to 9.1)	.04
T30	84.2 (15.1)	80.3 (16.5)	NA	NA
Mean change (95% CI)	−0.3 (−3.5 to 3.0)	−3.5 (−6.7 to −0.4)	3.2 (−1.3 to 7.7)	.16
T45	88.8 (15.8)	80.4 (15.8)	NA	NA
Mean change (95% CI)	1.1 (−8.6 to 10.8)	−4.7 (−10.4 to 1.1)	5.8 (−4.7 to 16.3)	.27
T60	86.0 (14.1)	76.6 (18.6)	NA	NA
Mean change (95% CI)	0.8 (−16.7 to 18.3)	−8.9 (−29.2 to 11.5)	NA	NA
GCS score, median (IQR)^c^				
T0	15.0 (15.0 to 15.0)	15.0 (15.0 to 15.0)	NA	NA
T15	15.0 (15.0 to 15.0)	15.0 (15.0 to 15.0)	NA	NA
Mean change (95% CI)	−0.2 (−0.3 to −0.1)	−0.02 (−0.04 to 0.01)	−0.2 (−0.3 to −0.1)	.01
T30	15.0 (15.0 to 15.0)	15.0 (15.0 to 15.0)	NA	NA
Mean change (95% CI)	−0.1 (−0.2 to 0.0)	0.0 (−0.1 to 0.0)	−0.1 (−0.2 to 0.0)	.07
T45	15.0 (15.0 to 15.0)	15.0 (15.0 to 15.0)	NA	NA
Mean change (95% CI)	−0.1 (−0.2 to 0.0)	−0.1 (−0.2 to 0.0)	0.0 (−0.2 to 0.1)	.61
T60	15.0 (15.0 to 15.0)	15.0 (15.0 to 15.0)	NA	NA
Mean change (95% CI)	−0.2 (−0.6 to 0.3)	0	NA	NA
Ramsay Sedation Scale score, median (IQR)^d^				
T0	2.0 (1.0 to 2.0)	2.0 (2.0 to 2.0)	NA	NA
T15	2.0 (2.0 to 3.0)	2.0 (2.0 to 2.0)	NA	NA
Mean change (95% CI)	0.5 (0.3 to 0.7)	0.2 (0.1 to 0.3)	0.3 (0.1 to 0.5)	.07
T30	2.0 (2.0 to 2.0)	2.0 (2.0 to 2.0)	NA	NA
Mean change (95% CI)	0.4 (0.2 to 0.6)	0.3 (0.2 to 0.4)	0.1 (−0.1 to 0.3)	.40
T45	2.0 (2.0 to 3.0)	2.0 (2.0 to 2.0)	NA	NA
Mean change (95% CI)	0.5 (0.1 to 0.9)	0.5 (0.3 to 0.7)	0.0 (−0.4 to 0.4)	.87
T60	2.0 (2.0 to 2.0)	2.0 (2.0 to 2.0)	NA	NA
Mean change (95% CI)	0.2 (−1.6 to 2.0)	0.1 (−0.2 to 0.4)	NA	NA

Abbreviations: GCS, Glasgow Coma Scale; NA, not applicable; T0, initial vital sign assessment; T15, vital sign assessment at 15 minutes; T30, vital sign assessment at 30 minutes; T45, vital sign assessment at 45 minutes; T60, vital sign assessment at 60 minutes.

^a^ Mean change is calculated as Tx – T0.

^b^ Calculated as ketamine minus morphine change.

^c^ Scores range from 3 (worst) to 15 (best).

^d^ Scores range from 1 (awake; agitated or restless [or both]) to 6 (asleep; no response to glabellar tap or loud auditory stimulus).

### Adverse Events Analysis

Emergency physicians documented all observed or reported adverse events encountered at 15-minute intervals until arrival at the receiving ED. Thirty minutes after administration of the study medication, the ketamine group had adverse events reported in 49 of 120 patients (40.8% [95% CI, 32.0%-49.6%]) and the morphine group had them reported in 19 of 113 (16.8% [95% CI, 10.4%-25.0%]; risk difference, 24.0% [95% CI, 12.8%-35.2%]) (Table 3). The most common adverse effects were emergence phenomenon (24 of 120 [20.0%]) in the ketamine group and nausea (12 of 113 [10.6%]) in the morphine group. We did not observe any increase in adverse events after 30 minutes of out-of-hospital pain management among patients with data available (5 of 40 [12.5%] in the ketamine group; 9 of 49 [18.4%] in the morphine group; *P* = .56). No patient experienced a severe adverse event requiring withdrawal from the study, and no patient required intervention to manage an adverse event.

**Table 3.** Frequency of Adverse Effects Observed by Study Group

Adverse effect	Patient group				Risk difference (95% CI), %
	Ketamine (n = 120)		Morphine (n = 113)		
	No. of patients	Risk (95% CI), %	No. of patients	Risk (95% CI), %	
Nausea	8	6.7 (2.2 to 11.1)	12	10.6 (2.9 to 16.3)	−3.9 (−11.2 to 3.3)
Vomiting	6	5.0 (1.1 to 8.9)	5	4.4 (0.6 to 8.2)	0.6 (−4.9 to 6.0)
Decreased consciousness (GCS score ≤13)^a^	8	6.7 (2.2 to 11.2)	3	2.7 (0.0 to 5.7)	4.0 (−1.4 to 9.5)
Visual disturbance	21	17.5 (10.7 to 24.3)	5	4.4 (0.6 to 8.2)	13.1 (5.3 to 20.9)
Emergence phenomenon^b^	24	20.0 (12.8 to 27.2)	1	0.9 (0.6 to 8.2)	19.1 (11.7 to 26.5)
Hypertension	5	4.2 (0.6 to 0.8)	1	0.9 (0.0 to 2.6)	3.3 (−0.7 to 7.3)
Total	49	40.8 (32.0 to 49.6)	19	16.8 (10.4 to 25.0)	24.0 (12.8 to 35.2)

Abbreviation: GCS, Glasgow Coma Scale.

^a^ Scores range from 3 (worst) to 15 (best).

^b^ Includes symptoms such as dysphoria, agitation, and hallucinations.

## Discussion

In this randomized clinical trial of patients with out-of-hospital traumatic pain, the use of intravenous ketamine compared with intravenous morphine showed noninferiority for pain relief, a conclusive result. These findings support the inference that there are no clinically meaningful differences between the analgesic effects of these 2 drugs and suggest that the use of intravenous ketamine represents an alternative to intravenous opioid analgesics for the treatment of adult patients with traumatic pain in an out-of-hospital setting.

A systematic review noted weaknesses in studies that assessed ketamine for the treatment of patients with acute pain in an out-of-hospital setting.^4^ Although the systematic review suggested that ketamine probably reduces pain more than opioids, the included studies had high risk of bias. As a result, in US and European guidelines, ketamine is not routinely recommended, and analgesic agents vary by country and according to the personnel providing care on the scene.^24,25^

Ketamine is a noncompetitive *N*-methyl-d-aspartate and glutamate receptor antagonist that decreases central sensitization, windup phenomena (ie, progressive increase of responses induced by repetitive nociceptive stimuli), and pain memory.^26,27,28^ Ketamine provides analgesic effects accompanied by preservation of protective airway reflexes, spontaneous respiration, and cardiopulmonary stability.^29,30^ Losvik et al^31^ reported that in patients with trauma and an injury severity score greater than 8, ketamine was associated with a significantly better effect on systolic blood pressure compared with opioids. Tran et al^17^ found that in patients with trauma, the mean effect, as measured by visual analogic score reduction, was 3.5 points for ketamine and 3.1 points for morphine (95% CI for difference, −0.8 to 0.09]). However, the results from these studies conducted in war zones are difficult to apply in civilian settings.^4^

Opioid prescription may be associated with severe adverse events, including oxygen desaturation and respiratory depression, hypotension, bradycardia, and oversedation, which can worsen a patient’s condition.^5,6,7^ Other common acute adverse effects of opioids include dizziness, nausea, and vomiting, which may limit morphine use in the out-of-hospital environment.^32^ Evidence also suggests that opioids may provide insufficient relief for out-of-hospital traumatic pain.^2^ In 2019, a survey on drug use from the US Department of Health and Human Services^33^ estimated that 10.1 million people 12 years and older had misused opioids in the previous year, and that 2 of 3 drug overdose deaths involved an opioid. Overdose deaths remain a leading cause of injury-related death in the US, and deaths involving synthetic opioids have increased in recent years.^34^ In an animal model, ketamine administration did not establish key addictionlike behavior and did not produce changes in the brain’s reward system linked to drug craving.^35^ Changing prescribing practices in acute pain management is a critical step in addressing the opioid epidemic and its adverse effects.^20^ With the growing potential for opioid addiction problems, the present study aimed to clarify the question by providing data on both the efficacy and rate of adverse events based on a direct comparison between intravenous ketamine and morphine, 2 treatments for out-of-hospital traumatic pain via a randomized clinical trial. Based on our findings, ketamine, which presents low addiction liability, is an alternative to opioids for adults with out-of-hospital traumatic pain and could help to mitigate the opioid crisis by reducing out-of-hospital opioid prescriptions.

### Limitations

This study has several limitations. First, the presence of a physician in the ambulance team may make the results of this study less relevant for US-based EMS systems where the number and training of available out-of-hospital clinicians clearly differ. Second, doses of morphine used in the trial were based on French recommendations, using small starting dose that may be less prone to provide rapid analgesia. Third, the single-blind design could have introduced performance bias. Fourth, we used a noninferiority design, but we were not able to show superiority in clinical secondary end points. Fifth, the follow-up time was limited to the out-of-hospital period, which limits the conclusions of the study, as patients in the ketamine arm may have been exposed to opioids during their subsequent hospital care.

## Conclusions

Among patients with out-of-hospital traumatic pain, the use of intravenous ketamine compared with morphine showed noninferiority for pain reduction in this randomized clinical trial, a conclusive result. We observed more adverse events in the ketamine group compared with the morphine group. These adverse events were minor and did not require intervention. This trial suggests the use of ketamine as an opioid-reduction alternative in an out-of-hospital setting to manage acute traumatic pain in adult patients.

## Notes

Supplement 1.
Trial Protocol and Statistical Analysis

Supplement 2. **eMethods.** Consent and Case Report Form Completion **eTable 1.** Number of Inclusions for Each Investigative Center **eTable 2.** Changes in Vital Signs on Enrollment and at 15-Minute Intervals Thereafter Until Arrival at the Receiving ED **eFigure**. Pain Severity Reduction by Trial Group on Enrollment and at 15-Minute Intervals Thereafter Until Arrival at the Receiving ED

Supplement 3.
Data Sharing Statement
