# Supplementary material for: Ketamine Compared With Morphine for Out-of-Hospital Analgesia for Patients With Traumatic Pain: A Randomized Clinical Trial
Source: JAMA Netw Open. 2024 Jan 29;7(1):e2352844. doi: 10.1001/jamanetworkopen.2023.52844 (PMC10825723; doi:10.1001/jamanetworkopen.2023.52844)
Supplement: Supplement 1. — Trial Protocol and Statistical Analysis [file jamanetwopen-e2352844-s001.pdf]

Supplementary Online Content

LE CORNEC C, LE POTTIER M, BROCH H, et al. Ketamine versus morphine for Out-of-Hospital Trauma Analgesia A Randomized Clinical Trial

Trial protocol

This supplementary material has been provided by the authors to give readers additional information about their work.

Ketamine versus morphine for Out-of-Hospital Trauma Analgesia: A Randomized Clinical Trial

Eudract : n° 2017-000930-69

Ref : RC17_0082

Ref CPP : 217 R26

BIOMEDICAL RESEARCH PROTOCOL

Version 4 du 22/05/2017
which has received the favourable opinion of the CPP (Institutional Review Board), the date, and the authorization of the ANSM

This biomedical research project will be funded by funding source

CENTRE HOSPITALIER
UNIVERSITAIRE DE NANTES

Sponsor:

Nantes University Hospital
Medical Affairs and Research Department
5, allée de l'île Gloriette
44 093 Nantes cedex 01 (FRANCE)
Tel: 33 (0)2 53 48 28 35
Fax : 33 (0)2 53 48 28 36

Coordinating researcher:

Emmanuel Montassier, emergency physician
Nantes University Hospital
02 53 48 20 38
emmanuel.montassier@chu-nantes.fr

Methodological support:

Jean-Benoit HARDOUIN
Institut de recherche en Santé 2 EA4275-SPHERE
18 boulevard Benoni-Goullin
44000 Nantes

Safety and Surveillance Unit for Clinical Research:

Dr Anne CHIFFOLEAU
Direction de la Recherche,
Département Promotion, Cellule Vigilances
CHU de Nantes,
5, allée de l'Ile Gloriette
44093 Nantes cedex 1
Tel : +33(0) 2 44 76 67 82
Fax : +33(0)2 53 48 28 36

This protocol will be designed and edited from version 4.0 of 22/05/2017 of the DIRC Nantes protocol type

HISTORY OF PROTOCOL AMENDMENTS

Version number (after amendment)	Date	Amendment justification
**Protocol version V4**	**22/05/207**	Initial version approved by IRB and ANSM
**Protocol version V5**	**23/09/2017**	Approved by IRB and ANSM. Aims of amendment:<br>- To allow use of paracetamol before injection of Ketamine or Morphine<br>- To correct a mistake in the packaging of Kétamine (use of vials of 50mg/5ml and not 250mg/5ml)<br>- To remove the unnecessary non-inclusion criteria “acute head injury and intracranial hypertension without controlled ventilation” since study requires a Glasgow score of 15 at inclusion.<br>- To indicate a change in principal investigator in hospital of Grenoble
**Protocol version V6** and its annexe1“List	**13/08/2018**	Approved by IRB. Aims of amendment:<br>- to add the participation of the

of investigators" and annexe 9 "summary of protocol		hospital of Gonesse to the study<br>- to increase the period of recruitment
**Annexe 1** "List of investigators" **version V4** of protocol V6	**28/01/2019**	Approved by IRB. Aims of amendment:<br>- to add the participation of the hospital of Bordeaux to the study<br>- To indicate a change in principal investigator in hospital of La Roche/Yon
**Annexe 9** " Summary of protocol" **version 7** of protocol V6	**17/04/2020**	Approved by IRB. Aims of amendment:<br>- to increase the period of recruitment until end of November 2022.

First patient was enrolled in the study on 23/11/2017 after approval of protocol V5 of 23/09/2017

MAIN CORRESPONDENTS OF THE STUDY

**<u>Coordinating Investigator</u>**<br>Emmanuel Montassier, emergency physician<br>Nantes University Hospital<br>02 53 48 20 38<br>emmanuel.montassier@chu-nantes.fr	**<u>Sponsor</u>**<br>Nantes University Hospital<br>Medical Affairs and Research Department<br>5, allée de l'île Gloriette<br>44 093 Nantes cedex 01 (FRANCE)<br>Tel: 33 (0)2 53 48 28 35<br>Fax : 33 (0)2 53 48 28 36
**<u>Methodology, Biostatistics</u>**<br>Jean Benoit Hardouin<br>Nantes University Hospital<br>& Institut de Recherche en Santé 2<br>Inserm 1260<br>18 boulevard Benoni-Goullin<br>44000 Nantes<br>jean-benoit.hardouin@univ-nantes.fr<br><br>**<u>Data Management Centre</u>**<br>Delegation for Clinical Research and Innovation (DRCI), Promotion & Quality Control<br>Nantes University Hospital	**<u>Coordinating pharmacy</u>**<br>Laurent Flet<br>Nantes University Hospital, Hôtel-Dieu<br>Pharmacy Department<br>1, place Alexis Ricordeau<br>44 093 Nantes cedex 01 (FRANCE)<br>Tel: 33 (0)2 40 08 41 54<br>laurent.flet@chu-nantes.fr

5, allée de l’île Gloriette<br>44 093 Nantes cedex 01 (FRANCE)<br>Tel: 33 (0)2 53 48 28 35<br>Fax : 33 (0)2 53 48 28 36	
**Coordination and monitoring**<br>Delegation for Clinical Research and Innovation (DRCI), Promotion & Quality Control<br>Nantes University Hospital<br>5, allée de l’île Gloriette<br>44 093 Nantes cedex 01 (FRANCE)<br>Tel: 33 (0)2 53 48 28 35<br>Fax : 33 (0)2 53 48 28 36	**Clinical research study vigilance unit**<br>Dr Anne CHIFFOLEAU<br>Direction de la Recherche,<br>Département Promotion, Cellule Vigilances<br>CHU de Nantes,<br>5, allée de l'Ile Gloriette<br>44093 Nantes cedex 1<br>Tel : +33(0) 2 44 76 67 82<br>Fax : +33(0)2 53 48 28 36

SIGNATURE PAGE

LIST OF ABBREVIATIONS

ADR	Adverse Drug reaction
AE	Adverse Event
ANSM	Agence Nationale de Sécurité des Médicaments et des produits de santé
AMM	Autorisation de Mise sur le Marché
CPP	Comité de Protection des Personnes
CNIL	Commission Nationale de l'Informatique et des Libertés
CRA	Clinical Research Assistant
DSMB	Data Safety Monitoring Board
DSUR	Development Safety Update Report
e-CRF	Electronic Case Report Form
ED	Emergency Department
GCP	Good Clinical Practices
ICH	International Conference on Harmonisation
IP	Investigational Product
INSERM	Institut National de la Santé et de la Recherche Médicale
ITT	Intention to Treat
MR	Méthodologie de référence
REC	Research Ethics Committee
SAE	Serious Adverse Event
SmPC	Summary of Product Characteristics
SOP	Standard Operating Procedures

SUSAR	Suspected Unexpected Serious Adverse Reaction

TABLE OF CONTENTS

Background and rationale ***14***

Study rationale ***15***

Study design ***16***

Patient Population ***18***

Study Intervention ***18***

Objectives ***19***

Main objective **20**

Secondary objectives **20**

Outcomes ***20***

Primary outcome **20**

Secondary outcomes **20**

Eligibility criteria ***20***

Inclusion criteria **21**

Non-inclusion criteria **21**

Sample size ***22***

Descriptive analyses ***22***

Statistical analyses ***22***

Analysis of primary outcome ***23***

Analyses of secondary outcomes ***23***

Vital sign changes during out-of-hospital management **23**

Adverse events **23**

Subgroup analyses ***23***

Interim analysis ***23***

Prohibited concomitant care ***24***

Intervention delivery ***24***

Identification of all data sources not included in the medical record ***24***

Discontinuation and withdrawal ***24***

Criteria in respect of discontinuation of all or part of the study (excluding biostatistical considerations) ***25***

Role of the funding source ... ***26***

Data handling ... ***26***

Data collection ... **26**

Access to data ... 26

Source data and source document ... 27

Data collection tool ... **27**

Confidentiality of data ... **27**

Data management procedures ... **28**

Data validation ... **28**

Security and archival of data ... **29**

Evaluation of security ... ***29***

List of expected ARs ... **29**

Description of safety evaluation parameters ... **29**

Procedures and timing for the measurement, collection and analysis of the safety evaluation parameters ... **30**

Reporting of non-serious adverse events ... **30**

Procedures in place for the documentation and the reporting of serious adverse events ... **30**

Procedure to follow for the patient concerned by an event/reaction and reporting period ... **31**

Procedures in place for the documentation and the reporting of serious adverse events ... **31**

Quality control – Monitoring visits ... ***31***

Audit and inspection ... ***32***

Storage of documents and data at the end of the study ... ***32***

By the investigators: ... **32**

By the sponsor: ... **33**

Administrative, ethical, regulatory considerations ... ***33***

Information and consent forms ... ***34***

CNIL ... ***34***

Research ethics committee ... ***34***

Regulatory authorities ... ***34***

Protocol amendments ... ***35***

Registration ... ***35***

Study funding and Insurance ... ***35***

Dissemination policy ... ***35***

Authorship ... **35**

Communication of the results to participants ... **36**

Background and rationale

Pain is a common condition among prehospital patients [1]. In Australia, Jennings et al. reported that 34.5% of prehospital patients experienced pain, the majority presenting with traumatic or medical etiology (40.1% and 39.1%, respectively). Pain of a cardiac nature only accounted for 17.0% of presentations [2]. Rapid and efficient management of acute pain is pivotal in the prehospital setting. However, Jennings et al. found that a large percentage of patients arrived in the emergency department (ED) without significant pain reduction [2]. In France, Galinski et al. reported that, overall, 51% of the patients experienced pain relief during prehospital management, and that inadequate pain control is more frequent in patients with traumatic or gynecologic/obstetric pain [3].

Opioids are the most frequently prescribed analgesics in the prehospital setting [3, 4]. However, several issues should be highlighted. First, opioids are highly addictive, and some patients may develop opioid dependence, even if they are exposed to brief opioid treatments during inhospital pain management [5–7]. Second, opioids prescription may be associated with severe adverse events, including oxygen desaturation and respiratory depression, hypotension, bradycardia, and oversedation, that may worse a patient's condition [8, 9]. Other common acute side effects of opioids include dizziness, nausea, and vomiting [10]. Therefore, alternative non-opioid analgesia strategy, using agents at lower risk of dependence, should be proposed to manage pain in the prehospital setting [11].

Ketamine is a non-competitive N-methyl-D-aspartate and glutamate receptor antagonist that decreases central sensitization, "wind-up" phenomena, and pain memory [12–14]. Ketamine is commonly used at a dissociative dose for procedural sedation [15]. Used at a subdissociative dose (i.e., low-dose ketamine, 0.1 to 0.6 mg/kg and, most commonly, 0.3 mg/kg), ketamine provides analgesic effects, accompanied by preservation of protective airway reflexes, spontaneous respiration, and cardiopulmonary stability [14, 16, 17].

Relatively few studies have reported the use of low-dose ketamine alone for analgesia in the prehospital setting. Losvik et al. conducted a retrospective cohort study of trauma patients, in a low cost rural trauma system in Iraq [18]. They reported that in patients with Injury Severity Score > 8, ketamine will be associated with a significantly better effect on the systolic blood pressure compared to opioid analgesia (p = 0.03). Tran et al. performed a cluster randomized trial to compare the analgesic effects of ketamine and morphine in trauma patients, in a prehospital low-resource setting [19]. A total of 169 trauma patients will be treated outside hospital settings with ketamine (administered as slow intermittent intravenous injections of doses of 0.2–0. 3 mg/kg), while 139 patients will be treated with morphine (administered in one single intramuscular dose of 10 mg for adult patients and 5 mg for child casualties). Visual Analogue Scale (VAS) ratings will be measured by district physicians at the first in-field encounter before the administration of analgesic, and then by trained physicians and nurses at ED admission. The mean effect, as measured by VAS reduction, will be 3.5 points for ketamine and 3.1 points for morphine (95% CI for a difference of − 0.8–0.09). The rate of vomiting will be significantly lower in the ketamine group (5%) than in the morphine group (19%, 95% CI for difference 8– 22%). The rate of hallucinations and agitation will be higher in ketamine-treated patients (11%) than in the morphine-treated patients (1.5%, 95% CI for difference 4–16%).

Study rationale

To do methodological limitations of the previous studies, well-designed multicenter clinical studies to further examine the potential applicability and benefits of subdissociative-dose ketamine in the prehospital setting in trauma and non-trauma patients are needed.

In this context, we will carry out a randomized, controlled, open label multicenter trial to compare a subdissociative-dose ketamine alone to morphine alone to provide pain relief in the prehospital setting in patients with traumatic pain. Here, we hypothesize that ketamine 20 mg, titrated during a 30-min period with an objective of verbal rating scale pain score of 3 or less, will provide non-inferior

analgesia to morphine 3 mg, titrated during the same period, in a group of patients suffering moderate to severe pain in the prehospital setting.

Study design

This is a randomized non-inferiority trial comparing two treatments (morphine versus ketamine) used for prehospital pain management. The study is a single blind study (patient blinded) (patient).

Randomization will be defined without block but will be stratified by center. Numbered, opaque and sealed envelopes will be used in each ambulance for the assignment of the type of treatment (ketamine or morphine).

This study (KETAMORPH trial) is a prospective, randomized, parallel-group, controlled, single-blinded, nationwide, noninferiority multicenter study to compare the effect of intravenous ketamine alone with that of morphine alone in the treatment of moderate (verbal numeric rating score between 5 and 7) to severe (verbal numeric rating score of 8 or greater) traumatic pain before arrival at hospital (Figure 1). The study patients are blinded to intervention assignment, but the physicians conducting the pain management are not blinded. We perform a single-blind trial as side effects associated with ketamine can easily be observed (dizziness, mood change). Therefore, blinding may not be complete as it might be possible to determine arm during administration. Moreover, the primary outcome is be assessed by the patient using the verbal rating scale, without any possible intervention of the physician in charge of the patient. This study will involve 11 prehospital emergency medical services (EMS) centers in France. These centers are ambulance base stations equipped with 1 or more mobile intensive care units, consisting of an ambulance driver, a nurse, and an emergency physician as the minimum team. All EMS personnel included in this study have experience conducting randomized trials. French out-of hospital medical systems are 2-tiered EMS response systems with advanced life support responders, including trained emergency physicians attending the scene by ambulance. The Comité de Protection des Personnes Sud-Méditerranée 2 ethics committee approved the trial protocol (ref IRB sudmed 2,

approval number 217 R26). Patients with out-of-hospital trauma with moderate to severe pain are most often not able to provide informed consent, because patients need urgent pain management and because acute pain impairs the ability to provide informed consent. Whenever a patient will be included without written informed consent, such consent will be promptly sought, according to the French Law of Ethics, subsequently from the patient when the pain has decreased. This study is registered at ClinicalTrials.gov (NCT03236805).

	**STUDY PERIOD**						
	**Enrolment**	**Allocation**	**Post-allocation**				**Close-out**
**TIMEPOINT****	$-t_1$	**0**	*15 min*	*30 min*	*45 min*	*ED admission*	***Hour 24***
**ENROLMENT:**							
**Eligibility screen**	X						
**Informed consent**	X or waived if pain impairs the ability to provide informed consent						
**Allocation**		X					
**INTERVENTIONS:**							
[Ketamine]		X	If needed	If needed	If needed	If needed	
[Morphine]		X	If needed	If needed	If needed	If needed	
**ASSESSMENTS:**							
numeric rating scale score		X	X	X	X	X	
vital signs		X	X	X	X	X	
rescue analgesia					X	X	
Adverse events			X	X	X	X	X
Rescue treatment			X	X	X	X	

Figure 1. Standard Protocol Items: Recommendations for Interventional Trials (SPIRIT) Figure for the KETAMORPH trial. Schedule of enrollment, interventions, and assessments.

Patient Population

Patients will be eligible for enrollment if they will be assessed by the attending EMS as having all of the following: aged 18 years or older, conscious (Glasgow Coma Scale [GCS] score=15), reporting traumatic pain with a verbal numeric rating scale pain score greater than or equal to 5 on a standard 11-point (0: no pain, to 10: worst possible pain) numeric rating scale, and speaking and able to rate their pain with the verbal numeric rating scale.

Patients will be excluded if any of the following applied: unstable vital signs (systolic blood pressure < 90 or > 200 mmHg, pulse rate < 50 or > 150 beats/min, and respiration rate < 10 or > 30 breaths/min, Glasgow Coma Scale score < 15), pregnancy, breast-feeding, unable to give numeric rating scale scores, allergy to morphine or ketamine, acute pulmonary edema or acute heart failure, acute coronary syndrome or unstable ischemic heart disease, renal or hepatic insufficiency, patients who received morphine for the same acute pain or acute psychiatric illness, patients who require emergency fracture or joint reduction, head injury with acute intracranial hypertension, patient using buprenorphine, nalbuphine, pentazocine or naltrexone.

Study Intervention

Patients will be randomized in a 1:1 ratio to the ketamine or the morphine group using a computer-generated list (Figure 1). Development of the randomization list, confirmation of written consent acquisition for all participants, and statistical analyses will be conducted by the research manager and statistician, who will be independent of any data collection. The randomization list will be generated before commencement of the study. We will used computer generated random numbers to generate the allocation sequence, without blocking. Numbered and sealed opaque

envelopes will be then generated from those lists and used by emergency physicians in each ambulance to assign patients to the morphine or ketamine group.

Morphine 10 mg will be diluted in 9 mL of normal saline solution, resulting in 1 mg/mL of solution. Morphine will be administered by intravenous push, 2 mg (patient weight < 60 kg) or 3 mg (patient weight ≥ 60 kg) every 5 min [ref]. Ketamine 200 mg will be diluted in 18 mL of normal saline solution, resulting in 10 mg/mL of solution. Ketamine will be administered by intravenous push of 20 mg followed by intravenous push of 10 mg every 5 min [ref]. Emergency physicians used their clinical judgment on dosing according to patient age and body size. Either morphine or ketamine continued to be administered according to this schedule until the patient became pain free (rating scale score of less or equal to 3), there will be a serious adverse event (eg, profound hypotension, unconsciousness, respiratory depression requiring ventilatory support), or the patient arrived at the receiving emergency department (ED). If a patient reports a pain numeric rating scale score of 5 or greater at 30 min, 45 min, 60 min or at ED admission, rescue analgesia will be administered to the patient for additional pain relief. The choice of drugs and dose will be left at the discretion of the emergency physician, as previously reported [ref]. For patients with a blood oxygen saturation level (SpO2) below 94% during the procedure, oxygen will be administered with nasal cannulae-delivering flow rate of 2 L/min, and will be adapted based on SpO2 follow-up.

Each physician will complete a paper case report form onsite. Later, to ensure the quality and completeness of the study data, a clinical research associate at each center verified the case report form data from the source medical file on-site and recorded the data to a centralized database. All 11 participating sites will complete identical case report form for each patient enrolled in the study.

Objectives

Main objective

The primary objective of the trial will to show that low-dose ketamine alone is not inferior to morphine alone at 30 min, in prehospital patients who experience moderate to severe, acute, traumatic or non-traumatic pain, defined as a numeric rating scale score greater or equal to 5.

Secondary objectives

Secondary endpoints will be:

- between-group difference in mean change in numeric rating scale pain scores among patients receiving ketamine or morphine, measured from the time before administration of the study medication to 15, 45, 60 min later, and at ED admission,
- the incidence of rescue analgesia at 30, 45, and 60 min, and at ED admission,
- the change in vital signs at 15, 45, 60 min and at ED admission,
- the incidence of adverse events at 15, 45, 60 min and at ED admission,
- the need to withdraw morphine or ketamine and the use of specific drugs to antagonize severe adverse events at 15, 45, 60 min and at ED admission,
- weight based dose of study drug (mg/kg dosing) received during the 30-min period,
- number of doses of study drug received during the 30-min period.

Outcomes

Primary outcome

The primary outcome will be the between group difference in mean change in verbal rating scale pain scores among patients receiving ketamine or morphine, measured from the time before administration of the study medication to 30 min later.

Secondary outcomes

Secondary endpoints will be:

- between-group difference in mean change in numeric rating scale pain scores among patients receiving ketamine or morphine, measured from the time before administration of the study medication to 15, 45, 60 minutes later, and at ED admission,

- the incidence of rescue analgesia,
- the change in vital signs at 15, 45, 60 minutes and at ED admission,
- the incidence of adverse events,
- the need to withdraw morphine or ketamine and the use of specific drugs to antagonize severe adverse events,
- the weight based dose of study drug (mg/kg dosing) received during the 30-min period

Eligibility criteria

Inclusion criteria

Patients will be eligible for enrollment if they will be assessed by the attending EMS as having all of the following:

- will be aged 18 years or older,
- conscious (Glasgow Coma Scale [GCS] score=15),
- reporting traumatic pain with a verbal numeric rating scale pain score greater than or equal to 5 on a standard 11-point (0: no pain, to 10: worst possible pain) numeric rating scale,
- and speaking and able to rate their pain with the verbal numeric rating scale.

Non-inclusion criteria

Patients will be excluded if any of the following applied:

- unstable vital signs
    - systolic blood pressure < 90 or > 200 mmHg,
    - pulse rate < 50 or > 150 beats/min,
    - respiration rate < 10 or > 30 breaths/min,
    - Glasgow Coma Scale score < 15,
- pregnancy,
- breast-feeding,
- unable to give numeric rating scale scores,
- allergy to morphine or ketamine,
- acute pulmonary edema or acute heart failure,
- acute coronary syndrome or unstable ischemic heart disease,
- renal or hepatic insufficiency,

- patients who received morphine for the same acute pain or acute psychiatric illness,
- patients who require emergency fracture or joint reduction,
- head injury with acute intracranial hypertension,
- patient using buprenorphine, nalbuphine, pentazocine or naltrexone.

Sample size

Hypotheses for sample size calculations integrated the results of 2 randomized clinical trials of this subject in the emergency department. These trials used a between-group difference for change in mean pain score of 1.3 to define a statistically difference. After assuming a noninferiority margin of 1.3, based on studies that focused on acute extremity pain in the emergency department using the same main outcome, with a type I error of 5%/2 and type II error of 10%, it will be determined that 112 patients will be needed in each group. We set targeted enrollment at 248 patients to take into account risks of protocol deviations in this emergency randomization context, considering 10% of non-evaluable subjects. Thus, we planned to include 124 patients in each group.

Descriptive analyses

Characteristics of patients in each group will be summarized in a descriptive table. Descriptive statistical analysis will include for each quantitative variable: the mean, the standard deviation, the minimums and maximums, as well as the median and the quartiles.

The qualitative variables will be expressed as frequencies and proportions. The standardized difference between the two groups will also be calculated for each variable and presented in this same table.

Statistical analyses

Analyses of the primary outcome and the secondary outcomes will be presented in a summary table. Qualitative variables will be presented as frequencies and proportions.

Quantitative variables will be presented as mean and standard deviation. The ordinal variables will be presented as median and quartiles. Analyses will be done using SAS software version 9.4.

Analysis of primary outcome

The non-inferiority between the difference in mean change in verbal rating scale pain scores among patients receiving ketamine or morphine, measured from the time before administration of the study medication to 30 minutes later.

The equivalence test will be a one-sided test based on the assumption of a non-inferiority margin of 1.3. The one-sided confidence interval at 97.5% of the difference will also be calculated using Wald's method.

This method allows control of Type I error in a non-inferiority setting. The analysis will be performed per protocol, as recommended for non-inferiority trials, and supplemented with an intention-to-treat analysis.

Analyses of secondary outcomes

Vital sign changes during out-of-hospital management

The comparison of proportions for each complication will be performed using a Chi2 test or an exact Fisher test according to the conditions of application.

Adverse events

The proportions of adverse events (serious and non-severe), their intensity, study imputation, and outcome will be described in a summary table, and compared between the two treatment arms, using a Chi2 or Fisher's exact test depending on the conditions of application.

Subgroup analyses

No subgroup analysis will be performed.

Interim analysis

No interim analysis is planned.

Prohibited concomitant care

No prohibited concomitant care, and based on up-to-date clinical practice guidelines and recommendations.

Intervention delivery

No run-ins and washouts periods or other specific aspect of time schedule of the intervention delivery will be made in the Ketamorph trial.

Identification of all data sources not included in the medical record

Data from the study may be compiled directly in the CRF. These data will not be reported in the source folder.

Discontinuation and withdrawal

Once a subject will be randomized in the study, every reasonable effort will be make to follow the subject for the entire study period even if there is a deviation from the intervention protocols, an early discontinuation of study treatment or if a participant misses one follow-up visit.

A subject may be discontinued from study treatment at any time if the subject, the investigator, or the Sponsor feels that it is not in the subject's best interest to continue. If a subject is withdrawn from treatment due to an adverse event, the subject will be followed and treated by the Investigator until the abnormal parameter or symptom has resolved or stabilized. All subjects who discontinue study treatment should be encouraged to complete all remaining scheduled visits and procedures.

Early discontinuation of study treatment is not a reason for withdrawal from the study.

All subjects are free to withdraw consent from participation at any time, for any reason, specified or unspecified, and without prejudice. Reasonable attempts will be made by the investigator to provide a reason for subject withdrawals. The reason for the subject's withdrawal from the study will be specified in the subject's source documents, in that event no further data will be collected for this participant, excepted the follow-up of ongoing serious adverse events, required by the patient's safety.

Nevertheless, data previously collected for this participant will be used. However, previous safety information which involved public health remained in sponsor anonymized data base.

Withdrawals from the study can only be effective after confirmation by the investigator and the sponsor. These withdrawals are always definitive.

Criteria in respect of discontinuation of all or part of the study (excluding biostatistical considerations)

The end of the study corresponds to the end of the collection of all the data necessary to the primary and secondary outcomes analysis, i.e. 6 months after the last visit of the last subject undergoing the trial.

A definitive or temporary discontinuation of all or part of the study may be decided by ANSM, the ERB.

In any case:

- A written confirmation of this early discontinuation of the study shall be sent to the coordinating investigator of the study (specifying the reasons for the early discontinuation) and to the principal investigator of each centre.
- All the patients included in the study shall be informed and should attend their early withdrawal visit.

Role of the funding source

The funding source will have no role in the study design, data collection, data analysis, data interpretation or writing of the report. All authors agreed to submit for publication.

Data handling

Data collection

Access to data

Prior to the trial initiation, study personnel will undergo training sessions on data collection and will be individually tested on data entry as well as outcome assessments. Study data will be collected and managed using Ennov clinical electronic data capture tools hosted at Nantes University Hospital. Ennov clinical is a secure, webbased application designed to support data capture for research studies, providing: (1) an intuitive interface for validated data entry; (2) audit trails for tracking data manipulation and export procedures; (3) automated export procedures for seamless data downloads to common statistical packages; and (4) procedures for importing data from external sources.

The investigator will prepare and maintain adequate and accurate source documents designed to record all observations and other pertinent data for each subject of the study.

The sponsor is responsible for obtaining the agreement of all the parties involved in the study in order to guarantee direct access in all the sites where the study is being conducted to source data, source documents and reports, so that he can control their quality and audit them.

The investigator is responsible for all information collected on subjects enrolled in this study. All data collected during the course of this study must be reviewed and verified for completeness and accuracy by the Investigator.

Source data and source document

Any original document or object helping to prove the existence or accuracy of a piece of information or fact recorded during the study is defined as a source document.

Data collection tool

Study personnel with their own access right to the study database, will enter/capture data from source documents corresponding to a subject into the protocol-specific electronic Case Report Form (eCRF).

Each person responsible for the filling of the eCRF will have to be identified in the table of delegations of responsibilities of each center (see investigator's file) and will have a "user" account with specific computer rights linked to his role.
All the information required by the protocol will be entered in an eCRF and an explanation will be provided for each missing piece of information. The data must be collected as they are obtained and transcribed into these forms in a clear manner.

If a correction is required for an eCRF, the time and date stamps track the person entering or updating eCRF data and create an electronic audit trail.

Confidentiality of data

In accordance with the legislative provisions in force (articles L.1121-3 and R.5121-13 of the French Public Health Code), people with direct access to source data will take all necessary precautions to ensure the confidentiality of information relating to study intervention, research studies and people taking part in them, particularly as regard to their identity and the results obtained. These people, such as investigators themselves, are subject to professional secrecy.

During the biomedical research study or when it is over, the information collected on the people taking part in it and forwarded to the sponsor by the investigators (or any other specialized staff member involved) will be made

anonymous. Under no circumstances may the uncoded names or addresses of the people concerned appear in it.

For coding subjects in the database or any study documents, the first letter of the first name and first letter of the last name of the subject will be recorded, accompanied by a code showing the order of inclusion of the subject in a centre.

The sponsor will ensure that each person taking part in the study has given his agreement in writing for access to the individual data concerning him which is strictly necessary for quality control of the study.

Data management procedures

Data management will be performed by the Data management plateform of the Delegation for Clinical Research and Innovation (DRCI) of Nantes University Hospital. An eCRF will be developed using Ennov Clinical. eCRF will be managed in agreement with the Standardized Operating Procedures (SOP) of the Data management plateform of the DRCI of Nantes University Hospital. Clinical Research Associate (CRA) in charge of the study will be trained to the eCRF and in charge of the investigator's training. Data will be entered in investigating centers through a secure web site, monitored by CRAs and queries will be edited by data managers, in agreement with a specified data management plan.

A data review will be done prior locking the database. The database will be locked in agreement with the SOPs of the Data management plateform of the Delegation for Clinical Research and Innovation (DRCI) of Nantes University Hospital and data will be extracted in a SAS format or other, according to statistical requirements. Raw data will be stored in a XML format.

Data validation

After data have been entered into the study database, a system of computerized data validation checks will be implemented and applied to the database on a regular basis. After inconsistencies review, queries are entered,

tracked, and resolved through the electronic data capture system directly (omissions and discrepancies will be forwarded to investigator and CRA for resolution).

The study database will be updated in accordance with the resolved queries. All changes will be documented.

Security and archival of data

The database is safeguarded against unauthorized access by established security procedures; appropriate backup copies of the database and related software files will be maintained.

Databases are backed up by the database administrator in conjunction with any updates or changes to the database.

Evaluation of security

List of expected ARs

Within the scope of this protocol, the expected ARs are associated with the study treatment and comparator, the protocol (procedures of the study) and auxiliary treatment.

All drugs involved in the study are used according to the indication of their authorization or according to professional guidelines. Consecutively the reference documents for ADR identification are the Summary Product Characteristics (SmPC). The drug related adverse reactions are most often related to their pharmacological properties and dose dependent; the most frequent are summarized below and, all reaction expected with treatment under study and its comparator are detailed in each SmPC.

Description of safety evaluation parameters

According to regulation, each AE/AR reported by the patient or identified by the investigator must be collected and reported to sponsor, as soon as he is aware, if it meets to seriousness criteria from inclusion of the subject, to the end of the participation.

Safety evaluation is a secondary objective and adverse effects of special interest are listed in §4.3.

Procedures and timing for the measurement, collection and analysis of the safety evaluation parameters

Any AR/AE whether expected or unexpected, serious or not, must be real-time collected in the study eCRF.

Reporting of non-serious adverse events

Non-serious adverse events or reactions must be reported in the e-CRF with their date of occurrence, a description, their intensity evaluation (using the classification provided in Appendix 3), outcome and duration, method of resolution, aetiology, causal relationship with special regard to the research and any decisions made.

Procedures in place for the documentation and the reporting of serious adverse events

All SARs/SAEs, whether expected or unexpected, must be reported immediately (from the day the investigator is becoming aware of the event) to the sponsor by the mean of the eCRF.

The information mentioned on the notification form present in the eCRF and on joined documents must be complete, accurate, clear (no abbreviation…) and coded (no name, address or hospital number).

Serious adverse events that do not need to be reported -include:

- Some circumstances requiring hospitalization that are not covered by the hospitalization / prolongation of hospitalization criterion related to the study inclusion and planned in the protocol,
- Admission for social or administrative reasons,
- Hospitalization for routine treatment or monitoring of the disease studied that is not related to the deterioration of the participant's condition,
- Hospitalization for medical or surgical treatment scheduled before the start of the research.

Pregnancy, overdose, misuse, medication errors or potential medication errors, quality defects should be reported by the investigator to the sponsor even if there is no adverse reaction associated.

Procedure to follow for the patient concerned by an event/reaction and reporting period

All events/reactions, serious or not serious, expected or unexpected, must be followed up until recovery, consolidation or death (event closed).

All SAE/SAR must be reported to the sponsor if it happens for a research participant:

- Since the consent signature date,
- During all the participant follow up period scheduled by the study
- After the end of the patient follow-up and without any time limit if the investigator becomes aware of a delayed adverse reaction (malformation, secondary cancer, etc.) possibly linked to the experimental treatment.

Procedures in place for the documentation and the reporting of serious adverse events

In accordance with the regulations, the promoter will declare any suspicion of SUSAR to the competent authorities according to the regulatory deadlines (without delay in the case of a death or life-threatening case, 15 days for the other criteria of seriousness).

Quality control – Monitoring visits

A clinical research associate appointed by the sponsor will regularly visit each study centre during the process of setting up the study, one or more times during the study depending on the frequency of inclusions, and at the end of the study. During these visits, the following aspects will be reviewed:

- informed consent,
- compliance with the study protocol and the procedures set out in it,
- quality of the data collected in the case report form: its accuracy, missing data, consistency of the data with the source documents (medical records, appointment diaries, the originals of laboratory results etc.),

- adequate management of medicinal products.

The on-site monitoring visits shall be organised after making arrangements with the investigator. The CRAs should be able to consult on each site:

- the enrolled patients' data compilation records,
- the patients' medical and nursing files,
- the investigator file,
- the treatment storage and dispensation place.

Each monitoring visit will be performed according to the monitoring plan and then, a monitoring report will be written.

The protocol has been classified according to the estimated level of risk for the patient taking part in the study. It shall be monitored as risk B (foreseeable risk similar to that of standard care).

Audit and inspection

Within the scope of this study, an inspection or audit may be conducted. The sponsor and/or participating centres should be able to provide inspectors or auditors with access to the data.

An audit may be performed at any time by people appointed by the sponsor who are independent of those responsible for the study. The aim of an audit is to ensure the good quality of the study, that its results are valid and that the law and regulations in force are being observed.

The investigators agree to comply with the requirements of the sponsor and the relevant authority for an audit or an inspection of the study.

The audit can apply to all stages of the study, from development of the protocol to publication of the results and filing the data used or produced in the study.

Storage of documents and data at the end of the study

The following documents relating to this study are archived in accordance with Good Clinical Practice:

By the investigators:

- For a period of 15 years following the end of the study:

- The protocol and any amendments to the protocol.
- The case record forms.
- The source files of participants who signed a consent form.
- All other documents and letters relating to the study.
- The original copies of informed consent forms signed by participants

At the end of the study, the investigator shall also receive a copy of the data for each patient in the investigator's centre sent by the sponsor.

The investigator is responsible for all these documents for the regulation period of archiving.

By the sponsor:

- For a period of 15 years following the end of the study:
- The protocol and any amendments to the protocol.
- The originals of the case record files.
- All other documents and letters relating to the study.
- Documents relating to serious adverse events

The sponsor is responsible for all these documents for the regulation period of archiving.

No removal or destruction may be carried out without the sponsor's agreement. At the end of the regulation archiving period, the sponsor will be consulted regarding destruction. All the data, all the documents and reports could be subject to audit or inspection.

Administrative, ethical, regulatory considerations

The sponsor and the investigator or investigators undertake to conduct this study in compliance with the principles of the "Declaration of Helsinki", international (ICH) and French good clinical practice regulations and guidelines (Règles de bonnes pratiques cliniques pour les recherches biomédicales portant sur des médicaments à usage humain) as well as European regulations and/or national laws and regulations relating to clinical trials.

The study will be conducted in accordance with this protocol. With the exclusion of emergency situations necessitating taking specific therapeutic actions, the investigator or investigators undertake to observe the protocol in all respects, in

particular as regards obtaining consent and the reporting and follow-up of serious adverse events.

This research is registered in the European EudraCT database under n° registration number in accordance with art. L1121.15 of the French Public Health Act.

Information and consent forms

The emergency physician in charge of the patient (investigator) agrees to provide the subject with clear and precise information about the protocol and request from him/her a written and signed consent form. The investigator shall give the subject a copy of the information form and consent form.

The investigator shall also sign and date the consent form. Both documents should be issued at least in duplicate hard copy format so that the patient and the investigator can each keep a copy. The investigator's original shall be placed in the investigator file. If the consent form is signed in duplicate, the investigator keeps the original and gives the copy to the subject.

CNIL

The data compiled during the trial may be processed electronically in compliance with CNIL requirements.

Research ethics committee

The protocol, informed consent form, subject information sheet will be reviewed and approved by a French ethic committee (CPP) prior to study initiation.

Regulatory authorities

The sponsor will send an authorization request to French health authority (ANSM).

Protocol amendments

Requests for substantial modifications should be addressed by the sponsor for approval or notification to ANSM and/or the Ethical Review Board concerned in compliance with the law and its implementing decrees.

The amended protocol should be a dated updated version.

Any amendments to the protocol must be made known to all the investigators participating in the study. The investigators undertake to comply with the contents.

Any amendment modifying the management of participants or the benefits, risks or constraints of the study, etc. will be the subject of a new Participant Information and Informed Consent form which must be completed and collected according to the same procedure as used for the previous one.

Registration

The study protocol will be registered on ClinicalTrials.gov before recruitment of the first trial participant. Recorded data will be updated regularly.

Study funding and Insurance

The sponsor shall fund the study and take out an insurance policy covering the financial consequences of its civil liability in compliance with the regulations.

Dissemination policy

Authorship

Any written or oral communication of the results of the study will be previously agreed by the coordinating investigator and, if necessary, by the scientific committee constituted for the study. Publications regarding projects financed by the French Ministry of Health must include the following statement: "This study was supported by a grant from the French Ministry of Health (programme acronym, year and registered number)".

A copy of the publication shall be delivered to Nantes University Hospital, the study sponsor, which shall necessarily be cited.

We will follow the Recommendations for the Conduct, Reporting, Editing, and Publication of Scholarly Work in Medical Journals (updated in December 2014) from the International Committee of Medical Journal Editors (ICMJE). All investigators not-cited in the authorship will be listed as non-author contributors.

Communication of the results to participants

In accordance with the law n° 2002-303 of 4th March 2002, participants will be informed, at their request, of the overall results of the study.

Statistical analysis plan

Study design 2
Objectives 2
  Main objective 2
  Secondary objectives 2
Outcomes 2
  Primary outcome 2
  Secondary outcomes 2
Eligibility criteria 2
  Inclusion criteria 2
  Non-inclusion criteria 3
Sample size 3
Descriptive analyses 4
Management of missing data 4
Statistical analyses 4
Analysis of primary outcome 4
Analyses of secondary outcomes 5
  Vital sign changes during out-of-hospital management 5
  Adverse events 5
Subgroup analyses 5
Interim analysis 5
Tables templates 5
Role of the funding source 6

Study design

This is a randomized non-inferiority trial comparing two treatments (morphine versus ketamine) used for prehospital pain management. The study is a single blind study (patient blinded) (patient).

Randomization was defined without block but was stratified by center. Numbered, opaque and sealed envelopes were used in each ambulance for the assignment of the type of treatment (ketamine or morphine).

Objectives

Main objective

The primary objective of the trial will to show that low-dose ketamine alone is not inferior to morphine alone at 30 min, in prehospital patients who experience moderate to severe, acute, traumatic or non-traumatic pain, defined as a numeric rating scale score greater or equal to 5.

Secondary objectives

Secondary endpoints will be: (1) between-group difference in mean change in numeric rating scale pain scores among patients receiving ketamine or morphine, measured from the time before administration of the study medication to 15, 45, 60 min later, and at ED admission, (2) the incidence of rescue analgesia at 30, 45, and 60 min, and at ED admission, (3) the change in vital signs at 15, 45, 60 min and at ED admission, (4) the incidence of adverse events at 15, 45, 60 min and at ED admission, (5) the need to withdraw morphine or ketamine and the use of specific drugs to antagonize severe adverse events at 15, 45, 60 min and at ED admission, (6) weight based dose of study drug (mg/kg dosing) received during the 30-min period, and (7) number of doses of study drug received during the 30-min period.

Outcomes

Primary outcome

The primary outcome will be the between group difference in mean change in verbal rating scale pain scores among patients receiving ketamine or morphine, measured from the time before administration of the study medication to 30 min later.

Secondary outcomes

Secondary endpoints will be: (1) between-group difference in mean change in numeric rating scale pain scores among patients receiving ketamine or morphine, measured from the time before administration of the study medication to 15, 45, 60 min later, and at ED admission, (2) the incidence of rescue analgesia at 30, 45, and 60 min, and at ED admission, (3) the change in vital signs at 15, 45, 60 min and at ED admission, (4) the incidence of adverse events at 15, 45, 60 min and at ED admission, (5) the need to withdraw morphine or ketamine and the use of specific drugs to antagonize severe adverse events at 15, 45, 60 min and at ED admission, (6) weight based dose of study drug (mg/kg dosing) received during the 30-min period, and (7) number of doses of study drug received during the 30-min period.

Eligibility criteria

Inclusion criteria

Patients were eligible for enrollment if they were assessed by the attending EMS as having all of the following:

- were aged 18 years or older,

- conscious (Glasgow Coma Scale [GCS] score=15),
- reporting traumatic pain with a verbal numeric rating scale pain score greater than or equal to 5 on a standard 11-point (0: no pain, to 10: worst possible pain) numeric rating scale,
- and speaking and able to rate their pain with the verbal numeric rating scale.

Non-inclusion criteria

Patients were excluded if any of the following applied:

- unstable vital signs
    - systolic blood pressure < 90 or > 200 mmHg,
    - pulse rate < 50 or > 150 beats/min,
    - respiration rate < 10 or > 30 breaths/min,
    - Glasgow Coma Scale score < 15,
- pregnancy,
- breast-feeding,
- unable to give numeric rating scale scores,
- allergy to morphine or ketamine,
- acute pulmonary edema or acute heart failure,
- acute coronary syndrome or unstable ischemic heart disease,
- renal or hepatic insufficiency,
- patients who received morphine for the same acute pain or acute psychiatric illness,
- patients who require emergency fracture or joint reduction,
- head injury with acute intracranial hypertension,
- patient using buprenorphine, nalbuphine, pentazocine or naltrexone.

Sample size

Hypotheses for sample size calculations integrated the results of 2 randomized clinical trials of this subject in the emergency department. These trials used a between-group difference for change in mean pain score of 1.3 to define a statistically difference. After assuming a noninferiority margin of 1.3, based on studies that focused on acute extremity pain in the emergency department using the same main outcome, with a type I error of 5%/2 and type II error of 10%, it was determined that 112 patients were needed in each group. We set targeted enrollment at 248 patients to take into account risks of protocol deviations in this emergency randomization context, considering 10% of non-evaluable subjects. Thus, we planned to include 124 patients in each group.

Population definition

Populations	
Population	Definition
Intention-to-treat (ITT)	All randomized patients will be analyzed, including those for whom the ethical and administrative criteria have not been verified (for these patients, the data will be deleted, and all data used to calculate the primary endpoint will be imputed).

Modified Intention-to-treat (mITT)	The following are removed from the mITT population:<br>- Patients who withdrew consent to participate<br>- Patient under guardianship<br>- Patient under 18 years old<br>- Patient admitted to ED before 30 minutes without primary endpoint measurement
Per Protocol (PP)	Removed from the PP population:<br>- Patients excluded from the mITT analysis<br>- Patients not meeting major inclusion/non-inclusion criteria<br>- Patients receiving rescue analgesia before T30<br>- Patients for whom the primary endpoint was not available<br>- Patients who did not receive the treatment assigned to them by randomization

Descriptive analyses

Characteristics of patients in each group will be summarized in a descriptive table. Descriptive statistical analysis will include for each quantitative variable: the mean, the standard deviation, the minimums and maximums, as well as the median and the quartiles. The qualitative variables will be expressed as frequencies and proportions. The standardized difference between the two groups will also be calculated for each variable and presented in this same table.

Management of missing data

Prior to the analyses, a completion of the missing data of primary outcome will be carried out, if necessary. Imputations will be made for the primary outcome by the average of the values of the patients in the same group. No imputations will be made for secondary endpoints. The hypothesis adopted regarding the mechanism of occurrence of the missing data will be a so-called Missing At Random (MAR) hypothesis.

Statistical analyses

Analyses of the primary outcome and the secondary outcomes will be presented in a summary table. Qualitative variables will be presented as frequencies and proportions. Quantitative variables will be presented as mean and standard deviation. The ordinal variables will be presented as median and quartiles. Analyses will be done using SAS software version 9.4.

Analysis of primary outcome

The non-inferiority between the difference in mean change in verbal rating scale pain scores among patients receiving ketamine or morphine, measured from the time before administration of the study medication to 30 minutes later will be tested using the confidence interval method. The confidence interval at 97.5% of the difference will be calculated using mixed linear regression adjusted on center as random effect. The upper bounds of these confidence intervals must not exceed the non-inferiority limit defined at 1.3. This method allows

control of Type I error in a non-inferiority setting. The analysis will be performed per protocol, as recommended for non-inferiority trials, and supplemented with a analysis on intention-to-treat population.

Analyses of secondary outcomes

Vital sign changes during out-of-hospital management

Secondary outcomes will be analyzed on modified intention-to-treat population. The comparison between the two treatment arms will be performed using a mixed logistic regression adjusted on center as random effect for proportions for binary variables, and mixed linear regression adjusted on center as random effect will be used for quantitative variables.

Adverse events

The proportions of adverse events (serious and non-severe), their intensity, study imputation, and outcome will be described in a summary table, and compared between the two treatment arms, using mixed logistic regression adjusted on center as random effect.

Subgroup analyses

No subgroup analysis will be performed.

Interim analysis

No interim analysis is planned.

Tables templates

The table templates are shown below.

Table 1. Demographic data and injury characteristics of patients.

**Characteristics**	**All patients (n=)**	**Ketamine Group (n=)**	**Morphine Group (n=)**
**Female, No. (%)**			
**Age, y**<br>Median (IQR)<br>Minimum, maximum			

Table 2. Vital sign changes during out-of-hospital management for pain by study group.

**Parameter**	**Ketamine Group (n=)**	**Morphine Group (n=)**	**Risk Difference (Ketamine-Morphine Group)**	**p-value**
**Pulse rate, mean beats/min**<br>T_0<br>T_{30}<br>Mean change*<br>95% CI				

Table 3. Frequency of adverse effects observed, by study group.

**Adverse Effect**	**Ketamine Group (n=)**			**Morphine Group (n=)**			**Risk Difference (Ketamine–Morphine Group)**	
	Frequency	Risk, %	95% CI	Frequency	Risk, %	95% CI	Risk Difference, %	95% CI
Nausea								

Role of the funding source

The funding source will have no role in the study design, data collection, data analysis, data interpretation or writing of the report. All authors agreed to submit for publication.
