# Supplementary material for: Ketamine Compared With Morphine for Out-of-Hospital Analgesia for Patients With Traumatic Pain: A Randomized Clinical Trial
Source: JAMA Netw Open. 2024 Jan 29;7(1):e2352844. doi: 10.1001/jamanetworkopen.2023.52844 (PMC10825723; doi:10.1001/jamanetworkopen.2023.52844)
Supplement: Supplement 2. — eMethods. Consent and Case Report Form Completion eTable 1. Number of Inclusions for Each Investigative Center eTable 2. Changes in Vital Signs on Enrollment and at 15-Minute Intervals Thereafter Until Arrival at the Receiving ED eFigure. Pain Severity Reduction by Trial Group on Enrollment and at 15-Minute Intervals Thereafter Until Arrival at the Receiving ED [file jamanetwopen-e2352844-s002.pdf]

Supplementary Online Content

Le Cornec C, Le Pottier M, Broch H, et al. Ketamine compared with morphine for out-of-hospital analgesia for patients with traumatic pain: a randomized clinical trial. *JAMA Netw Open*. 2024;6(1):e2352844.
doi:10.1001/jamanetworkopen.2023.52844

eMethods. Consent and Case Report Form Completion
eTable 1. Number of Inclusions for Each Investigative Center
eTable 2. Changes in Vital Signs on Enrollment and at 15-Minute Intervals Thereafter Until Arrival at the Receiving ED
eFigure. Pain Severity Reduction by Trial Group on Enrollment and at 15-Minute Intervals Thereafter Until Arrival at the Receiving ED

This supplementary material has been provided by the authors to give readers additional information about their work.

© 2024 Le Cornec C et al. *JAMA Network Open*.

eMethods. Consent and Case Report Form Completion

Process for obtaining consent

Informed consent may be waived at randomization because patients will need urgent pain management and because acute pain impairs the ability to provide informed consent. Whenever a patient is included without written informed consent, such consent will be promptly sought, according to the French Law of Ethics, from the patient when the pain has decreased. Therefore, the senior emergency physician from the emergency medical service in charge of the patient will obtain informed consent once the patient has arrived at the hospital. Therefore, for each patient included in the study, a senior emergency physician always obtained informed written consent from the patient. Then, a member of the research team of the prehospital and emergency department unit will follow the patient during the 24-hour follow-up.

Onsite case report form completion

Each physician completed a paper case report form onsite. Later, in order to ensure the quality and completeness of the study data, a clinical research associate at each center verified the case report form (CRF) data from the source medical file on-site and recorded the data to a centralized database. All 11 participating sites completed identical CRF for each patient enrolled in the study.

© 2024 Le Cornec C et al. *JAMA Network Open*.

eTable 1. Number of Inclusions for Each Investigative Center

Center	No. of inclusions (N = 251)
Nantes University Hospital, n (%)	146 (58)
Angers University Hospital, n (%)	40 (16)
Chateaubriant Hospital, n (%)	13 (5)
Bordeaux University Hospital, n (%)	12 (5)
Rennes University Hospital, n (%)	10 (4)
Tours University Hospital, n (%)	10 (4)
La Roche sur Yon Hospital, n (%)	8 (3)
Grenoble University Hospital, n (%)	7 (2.5)
Gonesse Hospital, n (%)	2 (1)
Le Mans Hospital, n (%)	2 (1)
Saint Nazaire Hospital, n (%)	1 (0.5)

© 2024 Le Cornec C et al. *JAMA Network Open*.

eTable 2. Changes in Vital Signs on Enrollment and at 15-Minute Intervals Thereafter Until Arrival at the Receiving ED

Parameter	Ketamine Group (n=120)		Morphine Group (n=113)	
	N	N miss	N	Nmiss
**Pulse rate, beats/min**				
T0	119	1	112	1
T15	116	4	109	4
Mean change T15*, 95% CI	116	4	109	4
T30	114	6	104	9
Mean change*, 95% CI	114	6	104	9
T45	31	3	39	2
Mean change T45*, 95% CI	31	3	39	2
T60	5	1	7	1
Mean change T60*, 95% CI	5	1	7	1
**Respiratory rate, breaths/min**				
T0	109	11	101	12
T15	101	19	97	16
Mean change T15*, 95% CI	99	21	94	19
T30	99	21	89	24
Mean change*, 95% CI	96	24	85	28
T45	25	9	33	8
Mean change T45*, 95% CI	25	9	31	10
T60	4	2	7	1
Mean change T60*, 95% CI	4	2	6	2
**Systolic blood pressure, mm Hg**				
T0	117	3	112	1
T15	113	7	107	6
Mean change T15*, 95% CI	111	9	107	6
T30	113	7	103	10
Mean change*, 95% CI	113	7	103	10
T45	30	4	36	5
Mean change T45*, 95% CI	29	5	36	5
T60	5	1	7	1
Mean change T60*, 95% CI	5	1	7	1
**Diastolic blood pressure, mm Hg**				
T0	117	3	112	1
T15	112	8	107	6
Mean change T15*, 95% CI	110	10	107	6
T30	113	7	103	10
Mean change*, 95% CI	112	8	103	10
T45	29	5	36	5
Mean change T45*, 95% CI	28	6	36	5
T60	5	1	7	1
Mean change T60*, 95% CI	5	1	7	1

© 2024 Le Cornec C et al. *JAMA Network Open*.

**GCS score**				
T0	119	**1**	111	2
T15	118	**2**	112	1
Mean change T15*, 95% CI	118	**2**	112	1
T30	117	3	109	4
Mean change*, 95% CI	117	3	109	4
T45	33	1	36	5
Mean change T45*, 95% CI	33	1	36	5
T60	6	0	8	0
Mean change T60*, 95% CI	6	0	8	0
**Ramsay score**				
T0	116	4	109	4
T15	116	4	109	4
Mean change T15*, 95% CI	116	4	109	4
T30	113	8	107	6
Mean change*, 95% CI	113	7	107	6
T45	30	4	40	1
Mean change T45*, 95% CI	30	4	40	1
T60	6	0	8	0
Mean change T60*, 95% CI	6	0	8	0

Number of patients per group for the secondary outcomes and missing data.
T_0, Initial vital sign assessment; T_{30}, vital sign assessment at 30 minutes
*Mean change T_x=change (T_x–T_0), x in minutes

© 2024 Le Cornec C et al. *JAMA Network Open*.

eFigure. Pain Severity Reduction by Trial Group on Enrollment and at 15-Minute Intervals Thereafter Until Arrival at the Receiving ED

Verbal numeric rating scale assessment (number of patients with available data reported at the bottom of the figure at baseline, 15-minute, 30-minute, 45-minute and 60-minute of the prehospital management).

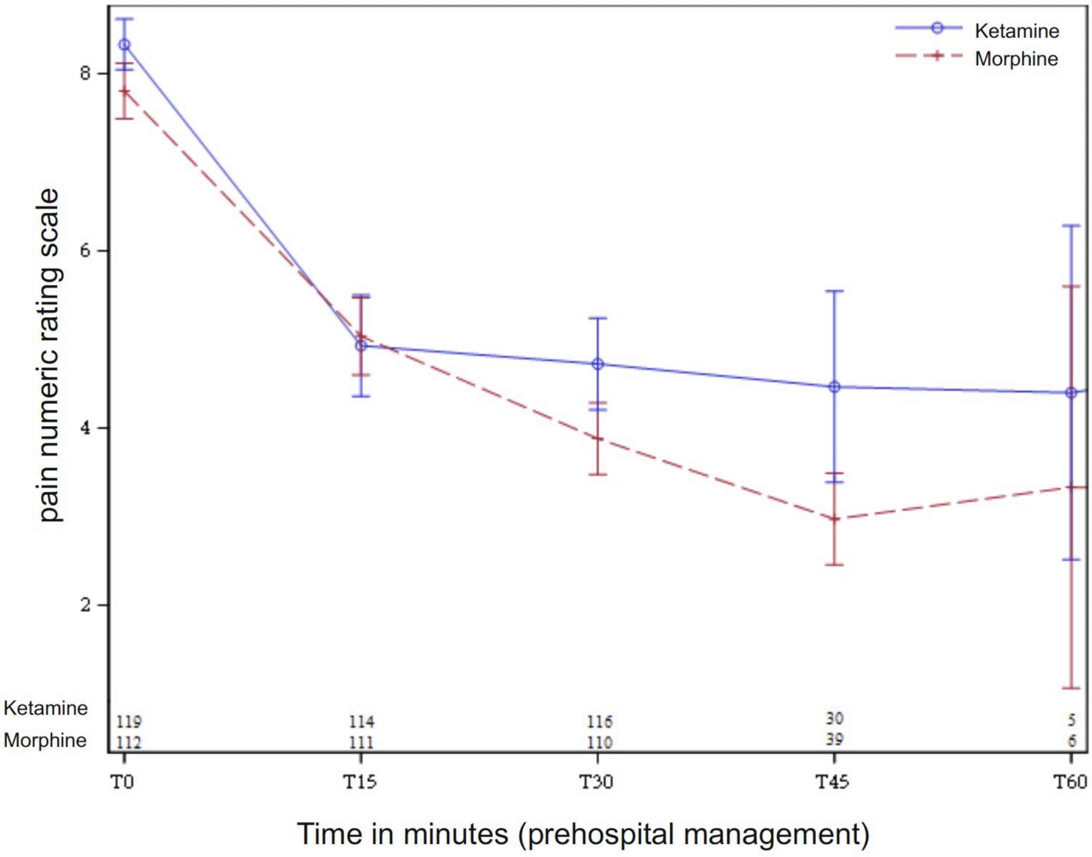

© 2024 Le Cornec C et al. *JAMA Network Open*.
