# Supplementary material for: Ketamine Compared With Morphine for Out-of-Hospital Analgesia for Patients With Traumatic Pain: A Randomized Clinical Trial
Source: JAMA Netw Open. 2024 Jan 29;7(1):e2352844. doi: 10.1001/jamanetworkopen.2023.52844 (PMC10825723; doi:10.1001/jamanetworkopen.2023.52844)
Supplement: Supplement 3. — Data Sharing Statement [file jamanetwopen-e2352844-s003.pdf]

# Data Sharing Statement

Le Cornec. Ketamine Compared With Morphine for Out-of-Hospital Analgesia for Patients With Traumatic Pain. *JAMA Netw Open*. Published January 29, 2024.
doi:10.1001/jamanetworkopen.2023.52844

**Data**
**Data available:** No

**Additional Information**
**Explanation for why data not available:** data available by contacting the PI
